# ﻿The fourth species of *Leptobrachella* (Anura, Megophryidae) found at Shiwandashan National Nature Reserve, Guangxi, China

**DOI:** 10.3897/zookeys.1192.98352

**Published:** 2024-02-22

**Authors:** Wei-Cai Chen, Peng Li, Wan-Xiao Peng, You-Jun Liu, Yong Huang

**Affiliations:** 1 Key Laboratory of Environment Change and Resources Use in Beibu Gulf Ministry of Education, Nanning Normal University, Nanning 530001, China; 2 Guangxi Key Laboratory of Earth Surface Processes and Intelligent Simulation, Nanning Normal University, Nanning 530001, China; 3 Shiwandashan National Nature Reserve, Fangcheng 538000, China; 4 Guangxi University of Chinese Medicine, Nanning 530200, China

**Keywords:** Bioacoustics, morphology, phylogeny, sympatric species

## Abstract

A new species of the genus *Leptobrachella*, *L.guinanensis***sp. nov.**, is described in this study based on morphological, molecular, and bioacoustic data. The species was discovered in the Shiwandashan National Nature Reserve in Shangsi County, Guangxi, China. Phylogenetically, *L.guinanensis***sp. nov.** is closely related to *L.ventripunctata*. However, there are distinct morphological differences between *L.guinanensis***sp. nov.** and *L.ventripunctata*, as well as three other sympatric species (*L.shangsiensis*, *L.shiwandashanensis*, and *L.sungi*). These differences include body size (SVL 30.5–32.5 mm in males; 38.7–41.8 mm in females in the new species vs 25.5–28.0 mm in males, 31.5–35.0 mm in females in *L.ventripunctata*), the absence of brown spots on the ventral surface (vs chest and belly creamy white with many scattered brown spots in *L.ventripunctata*), 1/3 toe webbing and wide toe lateral fringes (vs no toe webbing and no lateral fringes in *L.ventripunctata*), and distinct dermal ridges under toes (vs absent in *L.ventripunctata*). Furthermore, the dominant vocal frequencies of the new species range from 7.3 to 8.3 kHz, which is unique compared to other *Leptobrachella* species and represents the highest dominant frequencies ever recorded. The Shiwandashan National Nature Reserve is now home to four known sympatric species of *Leptobrachella*.

## ﻿Introduction

The Shiwandashan National Nature Reserve is situated in southern Guangxi, China, near the Sino-Vietnamese border, at coordinates 21°30'–22°08'N, 107°30'–108°30'E. Covering an area of 1,745 km^2^, the reserve exhibits an elevation range from slightly below 200 m to 1,462 m at the summit of Mt. Shuliangling. With a tropical monsoon climate, the reserve lies within the tropical mountain climate zone. The average annual temperature varies between 21.3 °C and 22.4 °C, while the total amount of annual precipitation ranges from 1,203.6 to 2,820.2 mm (Tan 2014). Recent literature reports the presence of 47 amphibian species within the reserve (Ren et al. 2018). Over the past decade, six new amphibian species have been discovered in this reserve. These include *Leptobrachellashangsiensis* Chen, Liao, Zhou & Mo, 2019 (Chen et al. 2019); *Leptobrachellashiwandashanensis* Chen, Peng, Pan, Liao, Liu & Huang, 2021 (Chen et al. 2021); *Nidiranashiwandashanensis* Chen, Peng, Li & Liu, 2022 (Chen et al. 2022a); *Occidozygashiwandashanensis* Chen, Peng, Liu, Huang, Liao & Mo, 2022 (Chen et al. 2022b); *Odorranafengkaiensis* Wang, Lau, Yang, Chen, Liu, Pang & Liu, 2015 (Wang et al. 2015); and *Zhangixaluspinglongensis* (Mo, Chen, Liao & Zhou, 2016) (Mo et al. 2016). Additionally, the previously recorded *L.sungi* (Lathrop, Murphy, Orlov & Ho, 1998) (Mo et al. 2008) brings the total number of *Leptobrachella* species in this reserve to three. In our recent study, we collected 14 specimens of *Leptobrachella* within the reserve and observed distinct differences to the known three species. Therefore, this study employs an integrative approach involving morphological, molecular, and bioacoustics analyses to identify and describe this newly discovered species.

## ﻿Materials and methods

### ﻿Sampling and morphological examination

**Figure 1.**
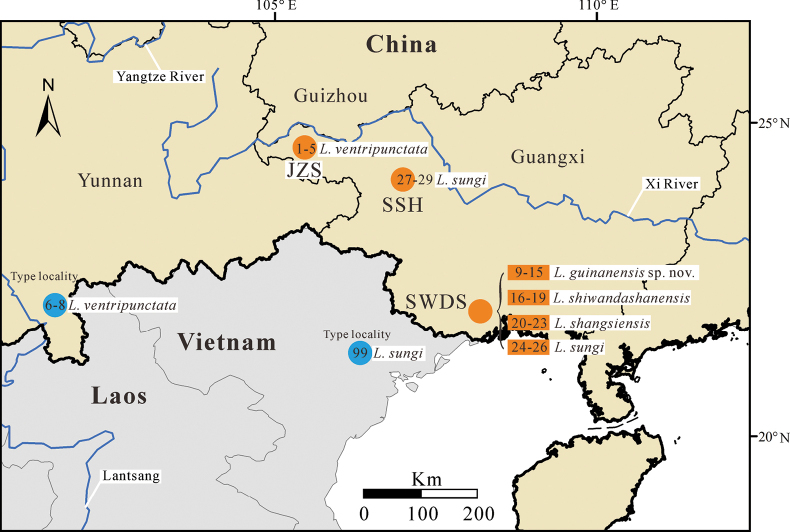
Localities of the new species and its sister taxa. Sample ID corresponding to those provided in Table 1.

Between 2021 and 2022, fourteen specimens were collected at the Shiwandashan National Nature Reserve (**SWDS**), Shangsi County, Guangxi, China (permission no. SWDS20210501). For comparison, nine specimens of *Leptobrachellaventripunctata* (Fei, Ye & Li, 1990) were collected at the Jinzhongshan National Nature Reserve on 22 June 2021 (JZS) (permission no. JZS20210605). Additionally, *L.sungi* specimens were collected at the SWDS (*n* = 16) on 4 July 2021, and the Sishuihe Nature Reserve (**SSH**) (*n* = 3) on 20 June 2020, located in Lingyun County, Guangxi, China (permission no. SSH20200615) (Fig. 1). After euthanasia using isoflurane, all specimens were fixed in 10% formalin for 48 h and finally stored in 75% ethanol. Muscle samples were taken prior to fixation and stored in 100% ethanol for subsequent molecular analyses. All specimens and muscle samples are deposited in the collection of Nanning Normal University (**NNU**) (see Table 1 for details). Specimens were measured with a digital caliper to the nearest 0.1 mm. The following measurements were taken:

**Table 1.** DNA sequences used in this study. ‘*’ represents type locality.

ID	Species	Locality	Voucher no.	16S
1	*L.ventripunctata*	Longlin County, Guangxi, China	NNU00527	OP548575
2	*L.ventripunctata*	Longlin County, Guangxi, China	NNU00528	OP548576
3	*L.ventripunctata*	Longlin County, Guangxi, China	NNU00529	OP548577
4	*L.ventripunctata*	Longlin County, Guangxi, China	NNU00530	OP548578
5	*L.ventripunctata*	Longlin County, Guangxi, China	NNU00531	OP548579
6	*L.ventripunctata*	Xishuangbanna, Yunnan, China*	SYS a001768	KM014811
7	*L.ventripunctata*	Xishuangbanna, Yunnan, China*	SYS a004539	MG520361
8	*L.ventripunctata*	Zhushihe, Xishuangbanna, Yunnan, China*	SYSa004536	MH055831
9	*L.guinanensis* sp. nov.	Shangsi County, Guangxi, China*	NNU00557	OP548561
10	*L.guinanensis* sp. nov.	Shangsi County, Guangxi, China*	NNU00558	OP548562
11	*L.guinanensis* sp. nov.	Shangsi County, Guangxi, China*	NNU00559	OP548563
12	*L.guinanensis* sp. nov.	Shangsi County, Guangxi, China*	NNU00560	OP548564
13	*L.guinanensis* sp. nov.	Shangsi County, Guangxi, China*	NNU00561	OP548565
14	*L.guinanensis* sp. nov.	Shangsi County, Guangxi, China*	NNU00569	OP548566
15	*L.guinanensis* sp. nov.	Shangsi County, Guangxi, China*	NNU00570	OP548567
16	*L.shiwandashanensis*	Fangcheng City, Guangxi, China*	NNU202103146	MZ326691
17	*L.shiwandashanensis*	Fangcheng City, Guangxi, China*	NNU202103213	MZ326692
18	*L.shiwandashanensis*	Fangcheng City, Guangxi, China*	NNU202103214	MZ326693
19	*L.shiwandashanensis*	Fangcheng City, Guangxi, China*	NNU202103215	MZ326694
20	*L.shangsiensis*	Shangsi County, Guangxi, China*	NHMG1401032	MK095460
21	*L.shangsiensis*	Shangsi County, Guangxi, China*	NHMG1401033	MK095461
22	*L.shangsiensis*	Shangsi County, Guangxi, China*	NHMG1704002	MK095462
23	*L.shangsiensis*	Shangsi County, Guangxi, China*	NHMG1704003	MK095463
24	*L.sungi*	Shangsi County, Guangxi, China	NNU00572	OP548569
25	*L.sungi*	Shangsi County, Guangxi, China	NNU00573	OP548570
26	*L.sungi*	Shangsi County, Guangxi, China	NNU00574	OP548571
27	*L.sungi*	Lingyun County, Guangxi, China	NNU00685	OP548572
28	*L.sungi*	Lingyun County, Guangxi, China	NNU00686	OP548573
29	*L.sungi*	Lingyun County, Guangxi, China	NNU00687	OP548574
30	*L.aerea*	Quang Binh, Vietnam	ZFMK 86362	JN848409
31	*L.alpina*	Caiyanghe, Yunnan, China	KIZ049024	MH055867
32	*L.applebyi*	Phong Dien Nature Reserve, Thua Thien-Hue, Vietnam	KIZ010701	MH055947
33	*L.arayai*	Borneo, Malaysia*	AE100/S9	DQ642119
34	*L.ardens*	Kon Ka Kinh National Park, Gia Lai, Vietnam*	ZMMU-NAP-06099	MH055949
35	*L.aspera*	Huanglianshan Nature Reserve, Lyuchun, Yunnan, China*	SYS a007743	MW046199
36	*L.baluensis*	Sabah, Borneo, Malaysia*	SP 21604	LC056792
37	*L.bashaensis*	Basha Nature Reserve, Guizhou, China*	GIB196404	MW136295
38	*L.bidoupensis*	Bidoup-Nui Ba National Park, Lam Dong, Vietnam*	ZMMU-A-4797-01454	MH055945
39	*L.bijie*	Bijie City, Guizhou, China*	SYS a007313	MK414532
40	*L.botsfordi*	Lao Cai, Vietnam*	AMS R 176540	MH055952
41	*L.bourreti*	Mao’er Shan, Guangxi, China	KIZ019389	MH055869
42	*L.brevicrus*	Sarawak, Borneo, Malaysia*	ZMH A09365	KJ831302
43	*L.chishuiensis*	Guizhou, China*	CIBCS20190518047	MT117053
44	*L.crocea*	Thua Thien-Hue, Vietnam	ZMMU-NAP-02274	MH055955
45	*L.damingshanensis*	Wuming County, Guangxi, China*	NNU202103281	MZ145229
46	*L.dorsospina*	Yushe Forest Park, Shuicheng, Guizhou, China*	SYS a004961	MW046194
47	*L.dringi*	Borneo, Malaysia*	KUHE:55610	AB847553
48	*L.eos*	Phongsaly, Laos*	MNHN 2004.0274	JN848452
49	*L.feii*	Yunnan, China*	KIZ048894	MT302634
50	*L.firthi*	Kon Tum, Vietnam*	AMS: R 176524	JQ739206
51	*L.flaviglandulosa*	Xiaoqiaogou Nature Reserve, Yunnan, China*	KIZ016072	MH055934
52	*L.fritinniens*	Danum Valley Field Center, Sabah, Malaysia	FMNH 244800	MH055971
53	*L.fuliginosa*	Phetchaburi, Thailand	KUHE:20197	LC201988
54	*L.gracilis*	Bukit Kana, Sarawak, Malaysia	FMNH 273682	MH055972
55	*L.graminicola*	Mount Pu Ta Leng, Lao Cai, Vietnam*	VNMN 010909	MZ224649
56	*L.hamidi*	Borneo, Malaysia*	KUHE 17545	AB969286
57	*L.heteropus*	Peninsular, Malaysia	KUHE 15487	AB530453
58	*L.isos*	Gia Lai, Vietnam*	AMS R 176480	KT824769
59	*L.itiokai*	Gunung Mulu National Park, Sarawak, Malaysia*	KUHE:55897	LC137805
60	*L.jinshaensis*	Lengshuihe Nature Reserve, Jinsha County, Guizhou, China*	CIBJS20200516001	MT814014
61	*L.juliandringi*	Sarawak, Borneo, Malaysia*	KUHE 17557	LC056784
62	*L.kajangensis*	Tioman, Malaysia*	LSUHC:4439	LC202002
63	*L.kalonensis*	Binh Thuan, Vietnam*	IEBR A.2014.15	KR018114
64	*L.kecil*	Cameron, Malaysia *	KUHE:52439	LC202003
65	*L.khasiorum*	Meghalaya, India*	SDBDU 2009.329	KY022303
66	*L.laui*	Wutongshan, Shenzhen city, China*	SYS a001507	KM014544
67	*L.liui*	Wuyi Shan, Fujian, China *	ZYCA907	MH055908
68	*L.macrops*	Dak Lak, Vietnam*	AMS R177663	KR018118
69	*L.maculosa*	Ninh Thuan, Vietnam*	AMS: R 177660	KR018119
70	*L.mangshanensis*	Manghan, Hunan, China *	MSZTC201703	MG132198
71	*L.maoershanensis*	Mao’er Shan, Guangxi, China	KIZ07614	MH055927
72	*L.marmorata*	Borneo, Malaysia*	KUHE 53227	AB969289
73	*L.maura*	Borneo, Malaysia	SP 21450	AB847559
74	*L.melanoleuca*	Kapoe, Ranong, Thailand	KIZ018031	MH055967
75	*L.melica*	Ratanakiri, Cambodia*	MVZ 258198	HM133600
76	*L.minima*	Doi Phu Fa, Nan, Thailand	KIZ024317	MH055852
77	*L.mjobergi*	Sarawak, Borneo, Malaysia*	KUHE 47872	LC056787
78	*L.murphyi*	Doi Inthanon, Chiang Mai, Thailand*	KIZ031199	MZ710523
79	*L.nahangensis*	Tuyen Quang, Vietnam*	ROM 7035	MH055853
80	*L.namdongensis*	Thanh Hoa, Vietnam*	VNUF A.2017.95	MK965390
81	*L.neangi*	Veal Veng District, Pursat, Cambodia*	CBC 1609	MT644612
82	*L.niveimontis*	Yongde County, Yunnan, China *	KIZ028276	MT302620
83	*L.nyx*	Ha GiangProv., Vietnam*	AMNH A 163810	DQ283381
84	*L.oshanensis*	Emei Shan, Sichuan, China*	Tissue ID: YPX37492	MH055896
85	*L.pallida*	Lam Dong, Vietnam*	UNS00510	KR018112
86	*L.parva*	Mulu National Park, Sarawak, Malaysia*	KUHE:55308	LC056791
87	*L.pelodytoides*	NA	TZ819	AF285192
88	*L.petrops*	Ba Vi National Park, Ha Tay, Vietnam	ROM 13483	MH055901
89	*L.picta*	Borneo, Malaysia	UNIMAS 8705	KJ831295
90	*L.pluvialis*	Lao Cai, Vietnam*	MNHN:1999.5675	JN848391
91	*L.puhoatensis*	Nghe An, Vietnam*	VNMN 2016 A.22	KY849586
92	*L.purpurus*	Yunnan, China *	SYSa006530	MG520354
93	*L.purpuraventra*	Guizhou, China *	SYSa007281	MK414517
94	*L.pyrrhops*	Loc Bac, Lam Dong, Vietnam*	ZMMU-A-4873-00158	MH055950
95	*L.rowleyae*	Da Nang City, Vietnam*	ITBCZ2783	MG682552
96	*L.sabahmontanus*	Borneo, Malaysia*	BORNEENSIS 12632	AB847551
97	*L.sola*	Gunung Stong, Kelantan, Malaysia	KU RMB20973	MH055973
98	*L.suiyangensis*	Guizhou, China *	GZNU20180606005	MK829649
99	*L.sungi*	Vinh Phuc, Vietnam *	ROM 20236	MH055858
100	*L.tadungensis*	Dak Nong, Vietnam*	UNS00515	KR018121
101	*L.tengchongensis*	Yunnan, China *	SYSa004598	KU589209
102	*L.tuberosa*	Kon Ka Kinh National Park, Gia Lai, Vietnam*	ZMMU-NAP-02275	MH055959
103	*L.wuhuangmontis*	Pubei County, Guangxi, China *	SYS a003486	MH605578
104	*L.wulingensis*	Hunan, China *	CSUFT194	MT530316
105	*L.yeae*	Mount Emei, Sichuan, China *	CIBEMS20190422HLJ1-6	MT957019
106	*L.yingjiangensis*	Yunnan, China *	SYSa006532	MG520351
107	*L.yunkaiensis*	Guangdong, China *	SYSa004663	MH605584
108	*L.zhangyapingi*	Chiang Mai, Thailand *	KIZ07258	MH055864
109	*Leptobrachiumhuashen*	Yunnan, China	KIZ049025	KX811931
110	*Xenophrysglandulosa*	Yunnan, China	KIZ048439	KX811762

**SVL** snout-vent length;

**HL** head length from the tip of snout to rear of jaws;

**HW** head width at commissure of jaws;

**SNT** snout length from the tip of snout to the anterior eye corner;

**ED** diameter of the exposed portion of eyeball;

**IOD** interorbital distance, the shortest distance between the anterior corners of the orbits;

**IN** internarial space distance;

**EN** distance from the eye to nostril, measured from the anterior corner of the eye to the posterior margin of the nostril;

**TD** horizontal diameter of tympanum;

**TED** distance from anterior edge of the tympanum to posterior eye corner;

**TIB** tibia length with flexed hindlimb;

**FLL** forelimb length from elbow to the tip of third finger;

**THL** thigh length from vent to knee;

**ML** manus length from the tip of third digit to proximal edge of the inner palmar tubercle;

**PL** pes length from the tip of fourth toe to the proximal edge of inner metatarsal tubercle;

**FEM** maximum diameter of femoral gland.

Sex was determined either directly through observation of calling males, presence of vocal sacs in males, or the presence of eggs in the abdomen of females. The webbing formula was determined following Savage (1975). Morphological data were obtained from the collected vouches specimens (Suppl. material 1: table S1) as well as other museum specimens (Suppl. material 1: table S2).

### ﻿Phylogenetic analyses

DNA was isolated from muscle samples using Tiangen Biotech Co. Ltd. tissue extraction kits (Beijing, China). The mitochondrial fragments of 16S (~530 bp) were amplified and sequenced using the primer pairs 16Sar_L (5’–CGCCTGTTTACCAA AAACAT–3’) and 16Sbr_H (5’–CCGGTCTGAACTCAGATCACGT–3’). Polymerase chain reaction (PCR) amplification followed the method described by Chen et al. (2021). The 16S fragments were sequenced on an ABI Prism 3730 automated DNA sequencer, and the new sequences were deposited in GenBank (OP548561–OP548567, OP548569–OP548579). Phylogenetic trees were reconstructed using the new sequences and homologous sequences of the genus *Leptobrachella* downloaded from GenBank (Table 1). The molecular data included topotypic sequences of *L.ventripunctata* (GenBank no. MH055831, KM014811, and MG520361) from Xishuangbanna, Yunnan, China (Sung et al. 2014; Chen et al. 2018; Yang et al. 2018). Bayesian inference (BI) and maximum likelihood (ML) methods were used to construct the phylogenetic trees. BI was performed using MrBayes v. 3.1.2 (Ronquist and Huelsenbeck 2003). The best-fit evolution model (GTR+I+G) was tested in JMODELTEST v. 2.1.7 (Posada 2008). Two independent runs with four Markov Chain Monte Carlo simulations were performed for 30 million iterations, and trees were sampled every 1,000^th^ generation. The first 25% of trees were discarded as burn-in. ML analysis was carried out on the CIPRES science gateway with 100 rapid bootstrap replicates (Miller et al. 2010) (https://www.phylo.org/portal2). Uncorrected *p*-distances of the 16S gene were estimated using Mega v. 7 (Kumar et al. 2016) with the default settings.

### ﻿Bioacoustics analysis

Advertisement calls were recorded using a SONY PCM-A10 recorder, and ambient temperature was measured using a digital hygrothermograph. The call recordings were analysed using the software Raven Pro v.1.6 (Cornell Laboratory of Ornithology, Ithaca, NY, USA). Audio-spectrograms were generated using Hanning windows, fast-Fourier transform (FFT) of 512 points, 50% overlap, and 172 Hz grid-spacing. Acoustic parameters were defined following Köhler et al. (2017) and Emmrich et al. (2020). Thus, we refer to a call refer as a group of notes, and the call duration is the time from the beginning of the first note to the end of the last note in a call. Call interval is defined as the time from the end of the last note of a call to the beginning of the first note of the subsequent call. Calls are often divided into two or more notes, which are smaller subunits that are usually separated by short intervals of silence relative to the note duration. The dominant frequency of a call is determined as the frequency with the highest energy concentration within the entire power spectrum.

### ﻿Morphological analysis

According to our results of the phylogenetic analyses, the new species is closely related to *L.ventripunctata*. Consequently Mann-Whitney U tests were conducted to determine the significance of differences in morphometric characters between the new species and *L.ventripunctata* (from JZS). Differences were considered significant below a threshold of 0.05. Principal component analysis (PCA) was performed to examine the distribution of the two species based on their morphometric parameters. Prior to the analysis, morphometric parameters were adjusted by calculating the ratio of each parameter to SVL, and then log-transformed to minimise the impact of allometry. All statistical analyses were carried out using IBM SPSS v. 20.

## ﻿Results

### ﻿Phylogenetic analyses and genetic divergence

**Figure 2.**
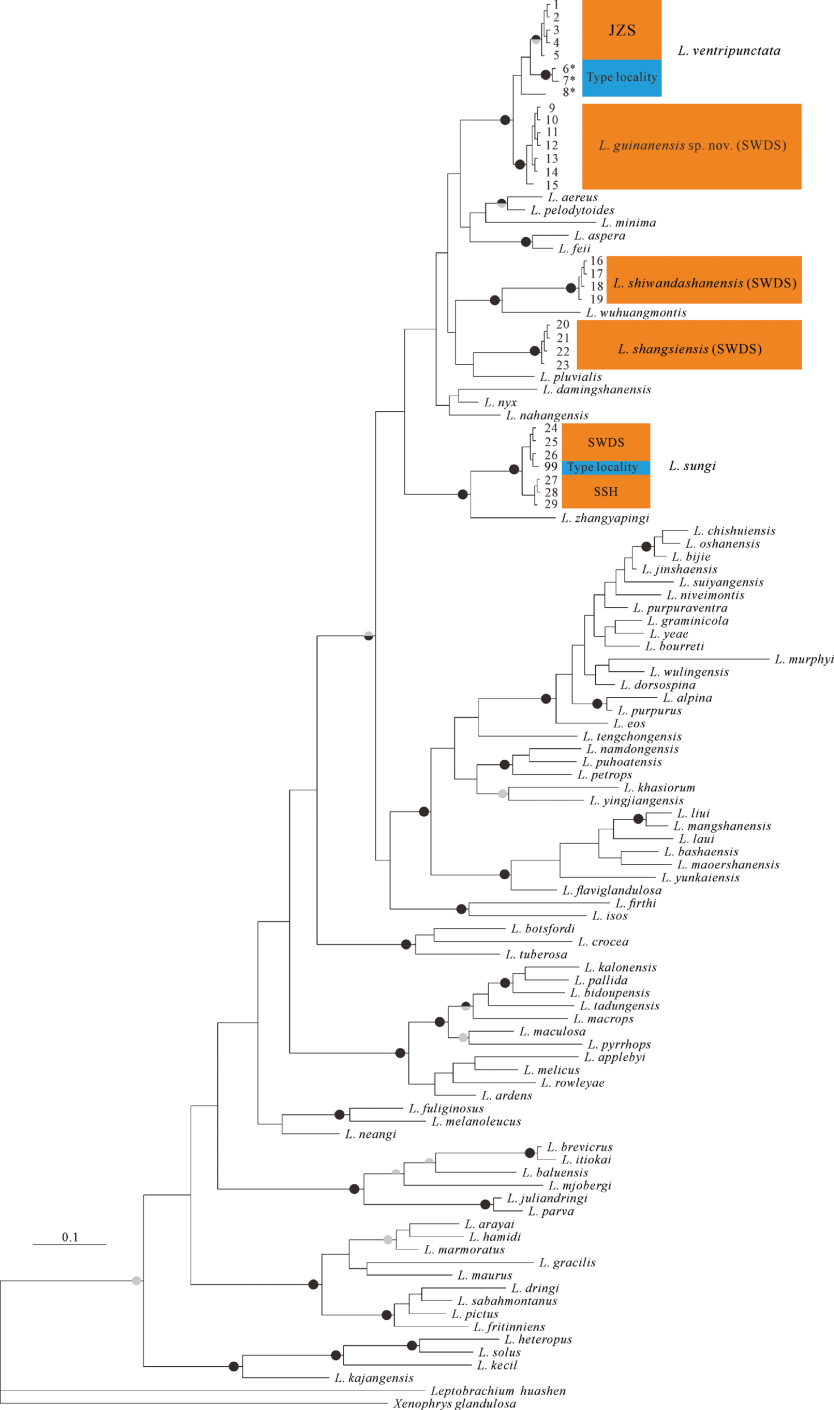
BI trees based on the part of the 16S gene. Node support is indicated on branches as maximum likelihood support (upper half; > 70% < 90% = grey, > 90% = black) and Bayesian posterior probabilities (lower half; > 0.95 = grey, 1 = black).

BI and ML analyses yielded nearly identical phylogenetic trees (Fig. 2). The preliminary phylogenetic trees revealed that all SWDS specimens were classified into four distinct lineages, corresponding to *L.shangsiensis*, *L.shiwandashanensis*, *L.sungi*, and an unidentified *Leptobrachella* lineage (Fig. 2). The newly collected specimens from SWDS formed a monophyletic group that is closely related to *L.ventripunctata*. The JZS specimens and *L.ventripunctata* from the type locality clustered together. The genetic divergences between the newly collected specimens and three sympatric species (*L.shangsiensis*, *L.shiwandashanensis*, and *L.sungi*) exceeded 8.2% (Suppl. material 1: table S3). The genetic divergences between the topotypic samples of *L.ventripunctata* and the newly collected specimens ranged from 1.6% to 2.4%, while those between the newly collected specimens and *L.ventripunctata* from JZS ranged from 1.7% to 1.9% (Suppl. material 1: table S3).

### ﻿Morphology

**Figure 3.**
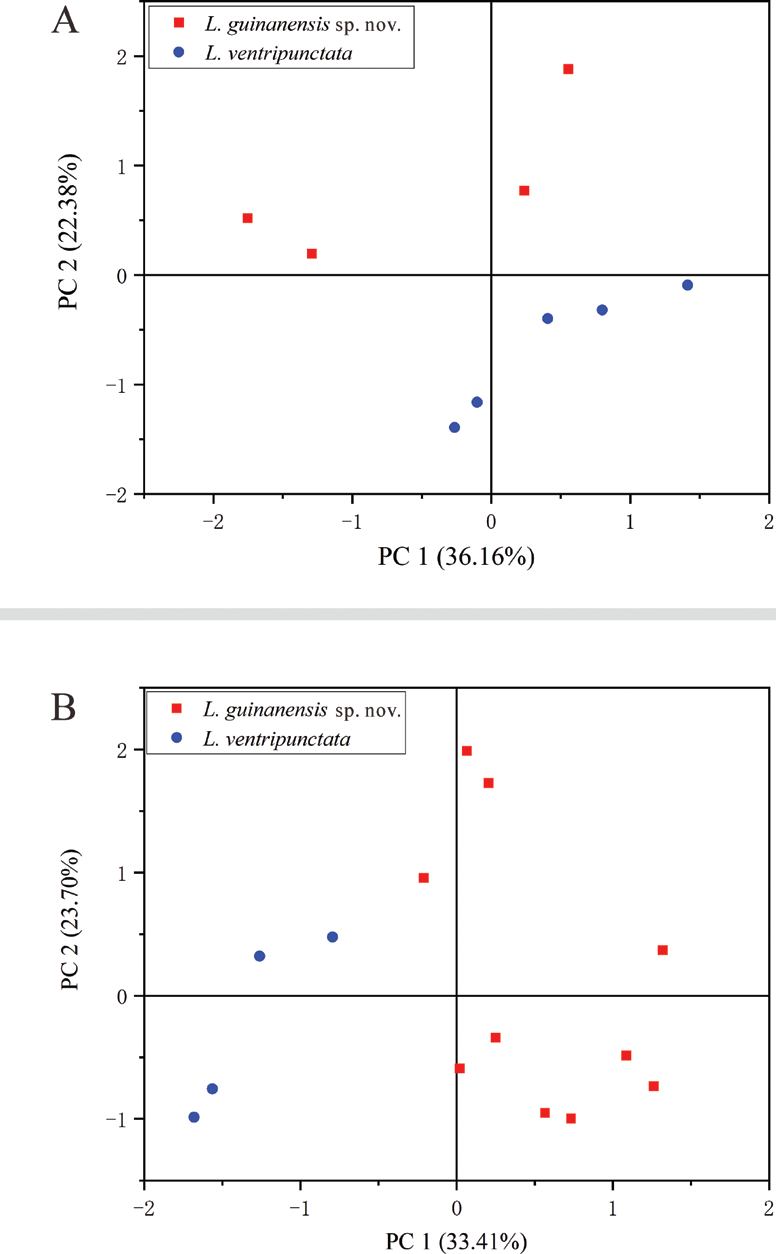
Scatter plot of PC1 and PC2 of PCA based on the morphometric measurements, distinguishing *L.guinanensis* sp. nov. and *L.ventripunctata***A** male comparison **B** female comparison.

The diagnostic characters for the new species of the genus *Leptobrachella* occurring north of the Isthmus of Kra are presented in Table 2, indicating that the newly collected specimens differ significantly from their congeners. The results of Mann-Whitney U tests revealed significant differences between the new specimens and *L.ventripunctata* from JZS in various measurements, including SVL, ED, IN, and FLL for males, and SVL, HL, HW, EN, TED, TIB, and PL for females (Table 3). There is no overlap in measurements between the new species and *L.ventripunctata* from JZS or the paratypes in terms of the measured parameters, including SVL, HL, HW, SNT, ED, TIB, FLL, ML, and PL (Table 3). Thus, PCA results showed clear differentiation between the new species and *L.ventripunctata* (Fig. 3). Furthermore, the newly described species exhibited distinct differences from *L.ventripunctata* in terms of body size (males being larger: SVL 30.5–32.5 mm vs 25.5–28.0 mm), ventral texture (ventral surface creamy white without dark brown spots vs chest and belly with dark brown spots), presence of a wide lateral fringe on toes (vs absence), presence of 1/3 toe webbing (vs absence), and presence of distinct dermal ridges under the toes (vs absence).

**Table 2.** Selected diagnostic characters for the species in the genus *Leptobrachella* occurring north of the Isthmus of Kra (modified from Rowley et al. 2017; Qian et al. 2020; and Wang et al. 2020). Toes webbing was determined following Fei et al. (2012). “Rudimentary” refers to an observable vestige of web.

ID	Species	Male SVL (mm)	Female SVL (mm)	Black spots on flanks	Toes webbing	Fringes on toes	Ventral colouration	Dorsal skin texture
1	*L.guinanensis* sp. nov.	30.5–32.5	38.7–41.8	Yes	One third	Wide	Ventral surface creamy white without dark brown spots	Dorsal surface shagreened with small, raised tubercles and longitudinal ridges
2	*L.aerea*	25.1–28.9	27.1–38.6	No	Rudimentary	Wide	Near immaculate creamy white, brown specking on margins	Finely tuberculate
3	*L.alpina*	24.0–26.4	31.7–32.1	Yes	Rudimentary	Wide	Creamy-white with dark spots	Relatively smooth, some with small warts
4	*L.applebyi*	19.6–22.3	21.7–26.4	Yes	Rudimentary	Absent	Reddish brown with white speckling	Smooth
5	*L.ardens*	21.3–24.7	24.5	Yes	Absent	Absent	Reddish brown with white speckling	Smooth-finely shagreened
6	*L.aspera*	22.4	25.0–26.4	Yes	Rudimentary	Narrow	Creamy white with distinct dark patches on chest and abdomen	Rough with dense conical granules, tubercles, and glandular folds
7	*L.bashaensis*	22.9–25.6	27.1	Yes	Rudimentary	Narrow	Creamy-white chest and off-white belly with irregular black spots	Dorsal skin slightly shagreened with small tubercles and irregular brown stripes
8	*L.bidoupensis*	18.5–25.4	28.3–29.4	Yes	Rudimentary	Narrow	Reddish brown with white speckling	Smooth
9	*L.bijie*	29.0–30.4	Unknown	Yes	Rudimentary	Narrow	White with distinct nebulous greyish speckling on chest and ventrolateral flanks	Shagreened and granular
10	*L.botsfordi*	29.1–32.6	30.0–31.8	No	Rudimentary	Narrow	Reddish brown with white speckling	Shagreened
11	*L.bourreti*	28.0–36.2	42.0–45.0	Yes	Rudimentary	Narrow	Creamy white	Relatively smooth, some with small warts
12	*L.chishuiensis*	30.8–33.4	34.2	Yes	Rudimentary	Narrow	White with distinct nebulous greyish speckling on chest and ventrolateral flanks	Shagreened and granular
13	*L.crocea*	22.2–27.3	Unknown	No	Rudimentary	Absent	Bright orange	Highly tuberculate
14	*L.damingshanensis*	33.6–34.4	Unknown	Yes	Rudimentary	Narrow	Creamy white ventral surface with small, creamy white glands on throat, chest and belly, becoming more concentrated near lateral margin	Rough dorsal skin with sparse jacinth tubercles and some short longitudinal ridges
15	*L.dong*	29.2–34.2	34.4–43.1	Yes	Rudimentary	Wide	White with distinct nebulous brown speckling on ventrolateral flanks	Shagreened with fine tubercles
16	*L.dorsospina*	28.7–30.5	32.1–39.8	Yes	Rudimentary	Narrow	Greyish white with black spots and orange pigmentations	Rough with dense conical granules, tubercles, glandular folds, and conical spines
17	*L.eos*	33.1–34.7	40.7	No	Rudimentary	Wide	Creamy white	Shagreened
18	*L.feii*	21.5–22.8	25.7	Yes	Rudimentary	Narrow	Creamy white with black blotches	Shagreened with small tubercles and ridge
19	*L.firthi*	26.4–29.2	25.7–36.9	No	Rudimentary	Wide	Creamy white	Shagreened with fine tubercles
20	*L.flaviglandulosa*	23.0–27.0	29.3	Yes	Rudimentary	Narrow	Whitish with black speckling on margins	Shagreened with yellowish-brown tubercles
21	*L.fuliginosa*	28.2–30.0	Unknown	Yes	Rudimentary	Narrow	White with brown dusting	Nearly smooth, few tubercles
22	*L.isos*	23.7–27.9	28.6–31.5	No	Rudimentary	Wide	Creamy white with white dusting on margins	Mostly smooth, females more tuberculate
23	*L.jinshaensis*	29.7–31.2	Unknown	Yes	Absent	Narrow	Ventral surface of throat cream white, chest, and belly cream yellow with purple speckling	Dorsal skin shagreened, some of the granules forming longitudinal short skin ridges
24	*L.jinyunensis*	29.1–34.1	34.1–34.9	Yes	Rudimentary	Narrow	Basically, floral white with deep grey pigments all over	Rough, covered with dense small granules and large tubercles
25	*L.kalonensis*	25.8–30.6	28.9–30.6	Yes	Absent	Absent	Pale, speckled brown	Smooth
26	*L.khasiorum*	24.5–27.3	21.2–33.4	Yes	Rudimentary	Wide	Creamy white	Isolated, scattered tubercles
27	*L.lateralis*	26.9–28.3	36.6	Yes	Rudimentary	Absent	Creamy white	Roughly granular
28	*L.laui*	24.8–26.7	28.1	Yes	Rudimentary	Wide	Creamy white with dark brown dusting on margins	Round granular tubercles
29	*L.liui*	23.0–28.7	24.5–27.8	Yes	Rudimentary	Wide	Creamy white with dark brown spots on chest and margins	Round granular tubercles with glandular folds
30	*L.macrops*	28.0–29.3	30.3	Yes	Rudimentary	Absent	Greyish violet with white speckling	Roughly granular with larger tubercles
31	*L.maculosa*	24.2–26.6	27.0	Yes	Absent	Absent	Brown with a few white speckling	Mostly smooth
32	*L.mangshanensis*	22.2–27.8	30.2	Yes	Rudimentary	Narrow	Throat grey-white and belly creamy white, scattered with white speckles	Smooth with orange tubercles and dark brown stripes
33	*L.maoershanensis*	25.2–30.4	29.1	Yes	Rudimentary	Narrow	Creamy white chest and belly with irregular black spots	Longitudinal folds
34	*L.melica*	19.5–22.7	Unknown	Yes	Rudimentary	Absent	Reddish brown with white speckling	Smooth
35	*L.minima*	25.7–31.4	31.6–37.3	Yes	Rudimentary	Absent	Creamy white	Smooth
36	*L.nahangensis*	40.8	Unknown	Yes	Rudimentary	Absent	Creamy white with light specking on throat and chest	Smooth
37	*L.namdongensis*	30.9	32.1–35.3	Yes	Rudimentary	Absent	Creamy white with brown dusting on margins	Finely tuberculate
38	*L.neangi*	Unknown (35.4–36.3 in females)	35.4–36.3	Yes	Rudimentary (in females)	Absent (in females)	Light purplish grey with dark brown mottling on throat	Small, irregular bumps and ridges
39	*L.niveimontis*	22.5–23.6	28.5–28.7	Yes	Rudimentary	Narrow	Marbling with black speckling	Relatively smooth with small tubercles
40	*L.nokrekensis*	26.0–33.0	34.0–35.0	Yes	Rudimentary	Unknown	Creamy white	Tubercles and longitudinal folds
41	*L.nyx*	26.7–32.6	37.0–41.0	Yes, but indistinct	Rudimentary	Absent	Creamy white with white with brown margins	Rounded tubercles
42	*L.oshanensis*	26.6–30.7	28.8–32.6	Yes	Absent	Absent	Whitish with no markings or only small, light grey spots	Smooth with few glandular ridges
43	*L.pallida*	24.5–27.7	Unknown	No	Absent	Absent	Reddish brown with white speckling	Tuberculate
44	*L.pelodytoides*	27.5–32.3	35.5–37.8	Yes	One third	Narrow	Whitish	Mostly smooth with smooth warts
45	*L.petrops*	23.6–27.6	30.3–47.0	No	Absent	Narrow	Immaculate creamy white	Highly tuberculate
46	*L.pingbianensis*	28.0	30.0	Yes	Rudimentary	unknown	Chest and belly with dark brown spots	Smooth
47	*L.pluvialis*	21.3–22.3	Unknown	Yes	Rudimentary	Absent	Dirty white with dark brown marbling	Smooth, ﬂattened tubercles on ﬂanks
48	*L.puhoatensis*	24.2–28.1	27.3–31.5	Yes	Rudimentary	Narrow	Reddish brown with white dusting	Longitudinal skin ridges
49	*L.purpuraventra*	27.3–29.8	33.0–35.3	Yes	Rudimentary	Narrow	Grey-purple with distinct nebulous greyish speckling on chest and ventrolateral flanks	Shagreened and granular
50	*L.purpurus*	25.0–27.5	Unknown	Yes	Rudimentary	Wide	Dull white with indistinct grey dusting	Shagreen with small tubercles
51	*L.pyrrhops*	30.8–34.3	30.3–33.9	Yes	Rudimentary	Absent	Reddish brown with white speckling	Slightly shagreened
52	*L.rowleyae*	23.4–25.4	27–27.8	Yes	Absent	Absent	Pinkish milk-white to light brown with white speckles	Smooth with numerous tiny tubercles
53	*L.shangsiensis*	24.9–29.4	30.8–35.9	Yes	Rudimentary	Narrow	Yellowish creamy white with marble texture	Smooth with numerous tiny tubercles
54	*L.shimentaina*	26.4–28.9	30.1–30.7	Yes	Rudimentary	Wide	Greyish pink with distinct hazy brown speckling on chest and ventrolateral flanks	Round granular tubercles with glandular folds
55	*L.shiwandashanensis*	26.8–29.7	33.7–35.9	Yes	Absent	Absent	Creamy white ventral surface with brown spots on lateral margin and near immaculate creamy white on throat and chest	Shagreened dorsal surface with small, raised tubercles and ridges, more evident on shoulder and dorsal surfaces of limbs
56	*L.suiyangensis*	28.7–29.7	30.5–33.5	Yes	Rudimentary	Narrow	Yellowish or creamy-white with marble texture or light brown speckling	Shagreened with small granules
57	*L.sungi*	48.3–52.7	56.7–58.9	No or small	Wide	Narrow	Yellowish or creamy-white	Granular
58	*L.tadungensis*	23.3–28.2	32.1	Yes	Absent	Absent	Reddish brown with white speckling	Smooth
59	*L.tamdil*	32.3	31.8	Yes	Wide	Wide	White	Weakly tuberculate
60	*L.tengchongensis*	23.9–26.0	28.8–28.9	Yes	Rudimentary	Narrow	White with dark brown blotches	Shagreened with small tubercles
61	*L.tuberosa*	24.4–29.5	30.2	No	Rudimentary	Absent	White with small grey spots/streaks	Highly tuberculate
62	*L.ventripunctata*	25.5–28.0	31.5–35.0	Yes	Absent	Absent	Chest and belly with dark brown spots	Longitudinal skin ridges
63	*L.verrucosa*	23.2–25.9	Unknown	Yes	Absent	Narrow	Creamy white with greyish white and dark brown spots	Shagreened with numerous conical tubercles
64	*L.wuhuangmontis*	25.6–30.0	33.0–36.0	Yes	Rudimentary	Narrow	Greyish white mixed with tiny white and black dots	Rough with dense conical tubercles
65	*L.wulingensis*	24.5–32.8	29.9–38.5	yes	Rudimentary	Narrow	Creamy white, with distinct or indistinct brown speckling at margins	Shagreened with sparse large warts, sometimes with longitudinal ridges
66	*L.yeae*	25.8–32.6	33.7–34.1	Yes	Rudimentary	Narrow	Ventral belly cream white with variable brown specking	Dorsum relatively smooth with fine tiny granules or short ridges
67	*L.yingjiangensis*	25.7–27.6	Unknown	Yes	Rudimentary	Wide	Creamy white with dark brown flecks on chest and margins	Shagreened with small tubercles
68	*L.yunkaiensis*	25.9–29.3	34.0–35.3	Yes	Rudimentary	Wide	Belly pink with distinct or indistinct speckling	Shagreened with short skin ridges and warts
69	*L.yunyangensis*	28.3–30.6	Unknown	Yes	Rudimentary	Narrow	Ventral surfaces of the throat, chest, and belly greyish white with purple-brown speckling	Rough dorsal skin, with sparse large granules and tubercles and short longitudinal ridges on the shoulder
70	*L.zhangyapingi*	45.8–52.5	Unknown	Yes	Rudimentary	Wide	Near immaculate white	Mostly smooth with distinct tubercles

**Table 3.** Morphometric measurements and comparisons between *L.guinanensis* sp. nov. and *L.ventripunctata*. “*” indicates *p*-value < 0.05; JZS = Jinzhongshan National Nature Reserve; SD = Standard deviation.

Characters	*p*-value from Mann-Whitney *U* test		*L.guinanensis* sp. nov.		*L.ventripunctata* (JZS)		*L.ventripunctata* (paratypes; Fei et al. 1992)
	Male	Female	Males (*n* = 4)	Female (*n* = 10)	Males (*n* = 5)	Females (*n* = 4)	Males (*n* = 10)
	New species vs *L.ventripunctata*	New species vs *L.ventripunctata*	Range (mean ± SD) (mm)	Range (mean ± SD) (mm)	Range (mean ± SD) (mm)	Range (mean ± SD) (mm)	Range (mean) (mm)
SVL	0.014*	0.005*	30.5–32.5 (31.8 ± 0.9)	38.7–41.8 (39.8 ± 1.5)	24.0–26.9 (26.0 ± 1.2)	32.0–34.5 (33.4 ± 1.1)	25.5–28.0 (26.5)
HL	0.806	0.011*	11.0–11.8 (11.3 ± 0.4)	14.0–15.3 (14.6 ± 0.4)	8.7–9.7 (9.3 ± 0.4)	11.1–12.0 (11.4 ± 0.4)	9.2–10.0 (9.6)
HW	0.142	0.048*	11.0–11.6 (11.4 ± 0.3)	14.0–15.5 (14.7 ± 0.6)	8.5–9.3 (9.0 ± 0.4)	11.1–12.5 (11.6 ± 0.6)	9.0–9.5 (9.5)
SNT	0.462	0.396	4.6–5.4 (4.9 ± 0.3)	5.1–6.5 (5.9 ± 0.4)	3.4–4.2 (3.8 ± 0.4)	4.6–4.9 (4.8 ± 0.1)	4.0–4.2 (4.1)
ED	0.014*	0.396	4.6–5.1 (4.9 ± .02)	5.2–5.9 (5.6 ± 0.2)	3.2–3.6 (3.5 ± 0.2)	4.3–5.1 (4.7 ± 0.4)	3.6–4.0 (3.8)
IOD	0.806	0.480	3.1–3.9 (3.5 ± 0.4)	3.7–4.3 (4.0 ± 0.3)	2.5–3.1 (2.9 ± 0.2)	2.9–3.2 (3.0 ± 0.1)	2.9–3.3(3.0)
IN	0.014*	0.258	3.4–4.1 (3.7 ± 0.3)	3.7–5.0 (4.2 ± 0.4)	2.3–2.8 (2.6 ± 0.2)	3.2–3.8 (3.6 ± 0.3)	Unknown
EN	0.086	0.016*	2.1–2.8 (2.5 ± 0.3)	2.9–3.3 (3.0 ± 0.1)	1.4–2.0 (1.8 ± 0.3)	2.2–2.4 (2.3 ± 0.1)	Unknown
TD	0.142	0.120	1.9–2.2 (2.0 ± 0.1)	1.7–2.9 (2.4 ± 0.4)	1.5–2.2 (2.0 ± 0.3)	2.0–2.7 (2.3 ± 0.4)	1.7–2.0 (1.8)
TED	0.624	0.048*	1.2–1.5 (1.3 ± 0.1)	2.0–2.8 (2.3 ± 0.3)	0.8–1.3 (1.1 ± 0.2)	1.3–1.8 (1.6 ± 0.2)	Unknown
TIB	0.327	0.005*	15.2–15.9 (15.5 ± 0.4)	18.5–19.4 (19.0 ± 0.3)	12.0–12.8 (12.3 ± 0.3)	13.9–15.2 (14.6 ± 0.5)	11.4–13.3 (12.1)
FLL	0.014*	0.480	14.4–15.4 (14.9 ± 0.5)	17.9–19.4 (18.8 ± 0.6)	12.3–13.3 (12.9 ± 0.4)	14.9–16.9 (15.5 ± 0.9)	12.1–14.2 (12.9)
THL	0.462	0.157	13.0–15.8 (14.5 ± 1.5)	18.2–19.6 (18.7 ± 0.5)	11.4–13.1 (12.2 ± 0.8)	14.6–16.6 (15.2 ± 0.9)	Unknown
ML	0.221	0.322	7.8–8.4 (8.1 ± 0.2)	9.3–10.2 (9.8 ± 0.4)	6.3–6.6 (6.4 ± 0.1)	7.1–8.7 (7.8 ± 0.7)	6.4–7.3 (7.0)
PL	1.000	0.032*	13.4–15.8 (14.4 ± 1.2)	15.4–19.0 (17.4 ± 1.5)	11.4–12.1 (11.7 ± 0.3)	12.8–14.7 (13.7 ± 0.9)	10.7–12.5 (11.4)
FEM	0.327	0.671	1.2–1.5 (1.3 ± 0.1)	1.4–2.2 (1.8 ± 0.3)	0.8–1.5 (1.2 ± 0.3)	1.2–1.6 (1.4 ± 0.2)	Unknown

### ﻿Bioacoustics

**Figure 4.**
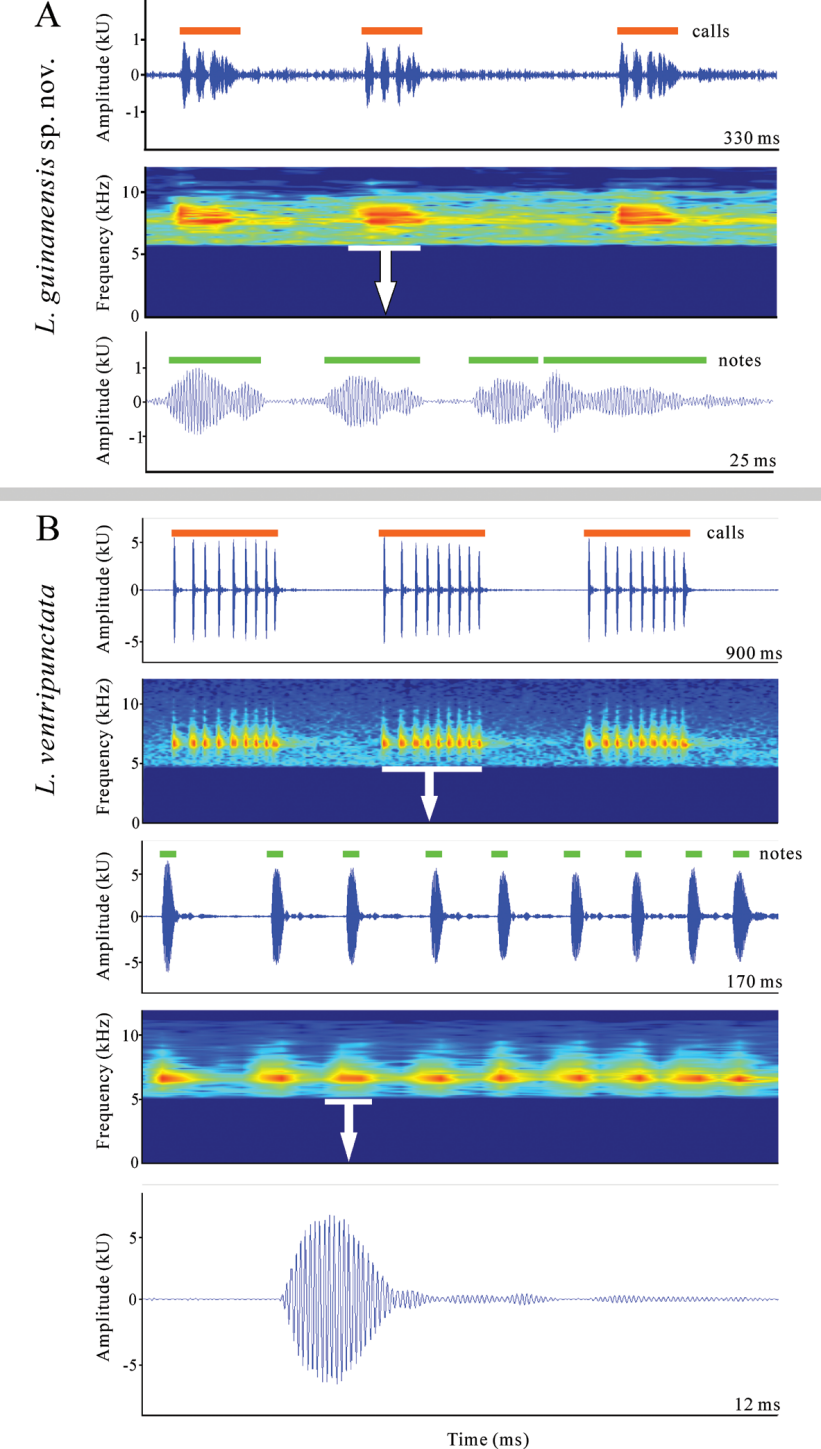
Advertisement calls of *L.guinanensis* sp. nov. **A** and *L.ventripunctata***B** including waveforms and spectrograms.

The calls of four individuals (NNU 00560–561, NNU 00875–876) were recorded. The main features of these calls are summarised in Table 4. The call of the newly described species consists of four notes (Fig. 4). The duration of the calls ranged from 23 milliseconds (ms) to 31 ms (mean 25.5 ± 1.4, *n* = 4), while the intervals between the calls were 55–133 ms (mean 91.2 ± 17.5, *n* = 4). The dominant frequencies of the calls were found to be in the range of 7.3–8.3 kHz. These characters were distinct from those of the sympatric species (*L.shangsiensis*, *L.shiwandashanensis*, and *L.sungi*) and *L.ventripunctata* (Fig. 4, Suppl. material 2: fig. S1, Table 4; Yang et al. 2018; Chen et al. 2019, 2021). Furthermore, the calls of the newly described species can also be differentiated from the known 40 species in the genus *Leptobrachella* (Suppl. material 1: table S4), as they possess the highest dominant frequencies ever recorded.

**Table 4.** Comparisons of characters of advertisement calls of the new species, sympatric species, and *L.ventripunctata*.

Species	Dominant frequency (kHz)	Call durations (ms)	Call intervals (ms)	Notes/call	Temperature (°C)	Reference
*L.guinanensis* sp. nov.	7.3–8.3	25.5 (23–31)	91.2 (55–133)	4–5	24.1	This study
*L.shiwandashanensis*	5.3–5.7	226.6 (194–277)	153.1 (134–186)	14–16	23.0	Chen et al. 2021b
*L.shangsiensis*	5.5–6.5	66.0 (64–69)	250.5 (184–289)	5–6	21.5	Chen et al. 2019
*L.sungi*	2.0–2.7	59.4 (56–65)	478.4 (225–996)	3	24.5	This study
*L.ventripunctata* (YJ)	6.1–6.4	145.0 (65–430)	134.0 (31–416)	3–17	15.0	Yang et al. 2018
*L.ventripunctata* (JZS)	6.2–7.1	182.8 (142–318)	215.7 (131–507)	8–9	25.1	This study

YJ, Yingjiang County, Yunnan, China; JZS, Jinzhongshan National Nature Reserve, Longlin County, Guangxi, Chin.

Based on the evidence from morphology, phylogeny, and bioacoustics, it is evident that the collected specimens represent a distinct, previously undescribed species within the genus *Leptobrachella*. Therefore, we describe these specimens as a new species of the genus *Leptobrachella*.

### ﻿Taxonomic account

#### ﻿ Leptobrachella guinanensis

Taxon classificationAnimaliaAnuraMegophryidae

Chen, Li, Peng & Liu sp. nov.

37AD6E83-0215-5F9E-B5DE-BBD0498C6F78

https://zoobank.org/412DCAC8-50F4-49D0-9F67-94D160AF1915

Figs 5 , 6

##### Material examined.

***Holotype*.**NNU 00876, adult male, collected at the Shiwandashan National Nature Reserve, Shangsi County, Guangxi, China (21°55'1.2"N, 107°54'10.8"E; elevation 512 m), collected by Wei-Cai Chen on 18 June 2022. ***Paratypes*.**NNU 00560–561, two adult males, NNU 00557–559, three adult females, collected at the same locality as the holotype on 10 June 2021; NNU 00569–571, three adult females, collected at the same locality as the holotype on 1 July 2021; NNU 00875, one adult male, NNU 00877–880, four adult females, collected at the same locality and time as the holotype. All specimens were collected by Wei-Cai Chen.

##### Etymology.

The species name *guinanensis* is derived from the geographic distribution of this species, specifically the southern Guangxi region. The suggested English name for this species is Gui Nan Leaf Litter Toad, while the Chinese name is Gui Nan Zhang Tu Chan (桂南掌突蟾).

##### Diagnosis.

*Leptobrachellaguinanensis* sp. nov. can be distinguished from its congeners by a combination of the following characters: (1) SVL 30.5–32.5 mm in males; 38.7–41.8 mm in females; (2) 1/3 toe webbing, wide lateral fringes; (3) dorsal surface shagreened with small, raised tubercles and longitudinal ridges; (4) ventral surface creamy white without dark brown spots; (5) throat immaculate creamy white and its margin concentrated brown spots; (6) iris bicoloured, upper half light copper, transitioning to silver in lower half; (7) crossbars of hindlimbs with tubercles; (8) distinct dermal ridges under the toes; (9) a pair of glands under the vent; (10) tibia-tarsal articulation reaching to centre of eye; (11) relatively higher dominant frequency of advertisement calls (7.3–8.3 kHz).

##### Description of holotype.

Adult male, SVL = 30.5 mm, head width less than length (HW/HL = 0.93); snout protruding, projecting over the lower jaw; nostril oval, closer to the tip of snout than eye; canthus rostralis distinct; loreal region sloping and slightly concave; interorbital region flat; pupil vertical; eye diameter near equal to snout length (ED/SNT = 0.99); tympanum distinct and rounded, and its diameter conspicuously less than eye diameter (TD/ED = 0.41); supratympanic fold distinct, raised from corner of eye to supra-axillary gland; vomerine teeth absent; vocal sac openings located laterally on the floor of mouth; tongue with a shallow notch at the posterior tip.

**Figure 5.**
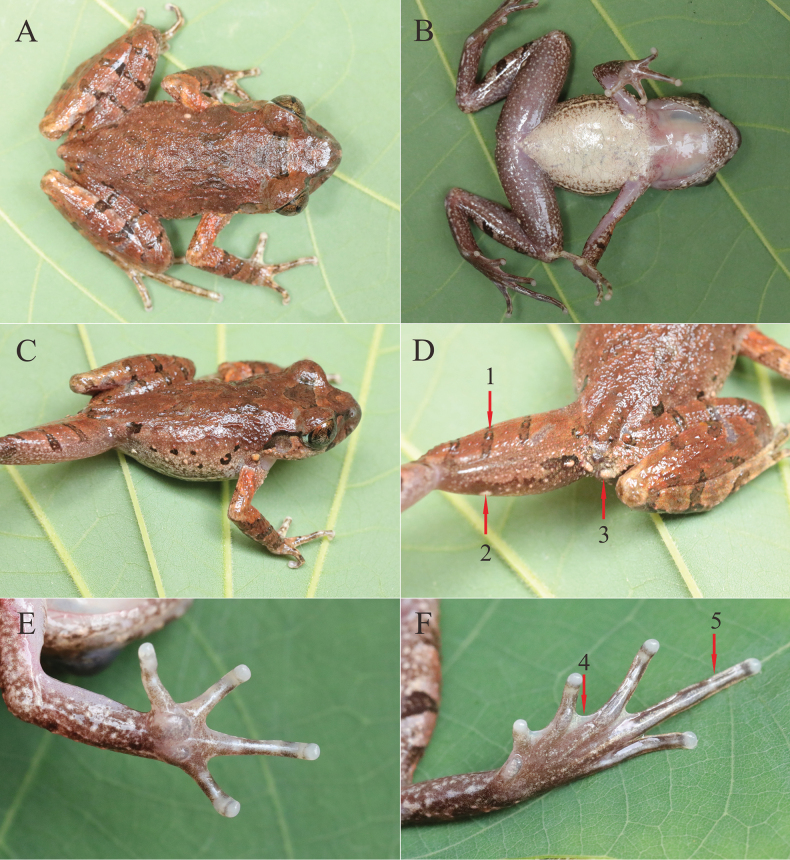
The holotype of *L.guinanensis* sp. nov. **A** dorsal view **B** ventral view **C** dorsolateral view **D** rear of the back and dorsal view of thighs **E** ventral view of hand **F** ventral view of foot. 1, tubercles on the crossbars; 2, femoral gland; 3, a pair of glands under the vent; 4, toe webbing; 5, wide lateral fringes on toe.

Tips of fingers rounded and slightly swollen; relative finger lengths I < II < IV < III; nuptial pad absent; subarticular tubercles absent; prominent inner palmar tubercle, separated from the small outer palmar tubercle; finger webbing and dermal fringes absent. Tips of toes rounded, slightly swollen; relative toe lengths I < II < V < III < IV; subarticular tubercles absent, replaced by distinct dermal ridges; pronounced large, oval inner metatarsal tubercle; outer metatarsal tubercle absent; 1/3 toe webbing; lateral fringes wide. TIB/SVL = 0.51; tibia-tarsal articulation reaching to the centre of eye; heels not meeting when thighs are appressed at right angles to body (Fig. 5).

Dorsal surface shagreened with small, raised tubercles and longitudinal ridges; belly and chest smooth without tubercles; anterior throat with several tubercles; ventral surface of limbs with creamy white tubercles; crossbars of hindlimbs with tubercles; flanks with several tubercles; pectoral glands oval, ~ 1.2 mm in diameter; femoral glands oval, ~ 1.3 mm in diameter, located on the posteroventral surface of thighs, closer to the knee than to the vent; supra-axillary glands distinct and rounded, ~ 0.9 mm in diameter; a pair of glands under the vent; and continued ventrolateral glandular line distinct (Fig. 5).

##### Colour of holotype in life.

Dorsal surface brown, an inverted triangle marking between eyes, irregular markings on shoulder and the rear of back; flanks with light orange tubercles; tympanum pale brown; supratympanic line black from posterior corner of eye to supra-axillary glands; posterior corner of eye silver; wide brown bars on upper lip; flanks with irregular black spots; brown transverse bars distinct on dorsal surface of forelimbs and hindlimbs; upper arm surfaces light orange; ventral surface creamy white without dark brown spots; throat immaculate creamy white and its margin concentrated brown spots; ventral surfaces of limbs purplish grey; pectoral and femoral glands, and a pair of creamy white glands under the vent, supra-axillary glands light orange; pupil black; iris bicoloured, upper half light copper, transitioning to silver in lower half (Fig. 5).

##### Colour of holotype in preservative.

Dorsum and limbs surfaces faded to a uniform grey; brown, inverted triangle marking distinctly visible between eyes; irregular black spots distinct on flanks; throat, chest, and belly creamy white; pectoral, femoral, supra-axillary, and ventrolateral glands creamy white; dark crossbars on limbs, fingers and toes remained distinct; upper arm and tibiotarsus faded to grey.

##### Variation.

**Figure 6.**
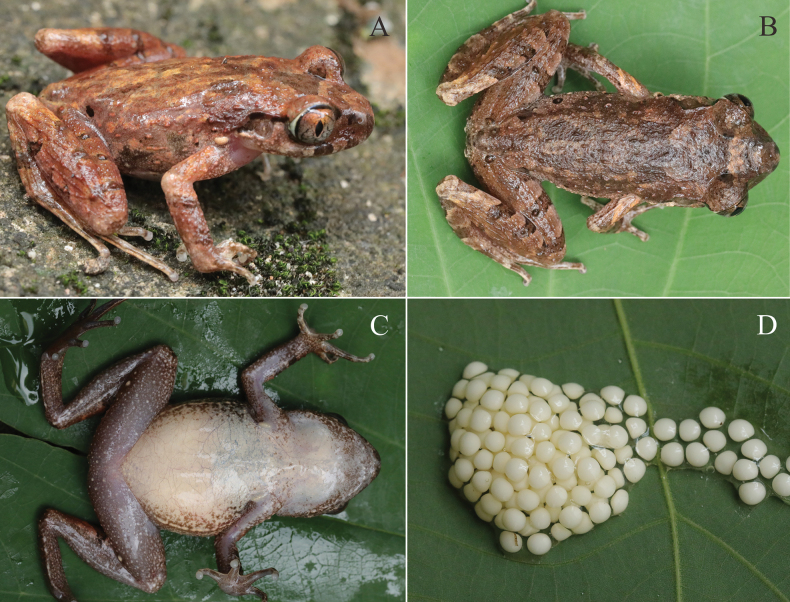
**A** more tubercles and longitudinal ridges on dorsum and hindlimbs surfaces (NNU00875) **B** light brown on dorsum (NNU00569) **C** ventral view of the gravid female (NNU00880) **D** eggs creamy white without black poles.

Measurements of the type series are provided in Table 3 and Suppl. material 1: table S5. The black spots and tubercles on the flanks exhibit variation between individuals. Certain individuals possess more tubercles and longitudinal ridges on their dorsum and hindlimb surfaces (Fig. 6A), while others display a light brown colouration on their dorsum (Fig. 6B).

##### Ecology and distribution.

*Leptobrachellaguinanensis* sp. nov. was discovered in the evergreen forest at SWDS, at an elevation of 400–600 m. The individuals were observed near rocky streams between 20:00–24:00 h. Males were found calling while sitting on rocks near the stream ~ 0.5–1.0 m. Females were found to be gravid with creamy white eggs (Fig. 6C) and laid their eggs in a bag while being raised indoors (Fig. 6D). Currently, *L.guinanensis* sp. nov. is only known from SWDS. So far within this reserve, we have identified four species of *Leptobrachella*, namely *L.guinanensis* sp. nov., *L.shangsiensis*, *L.shiwandashanensis*, and *L.sungi*.

##### Comparison.

**Figure 7.**
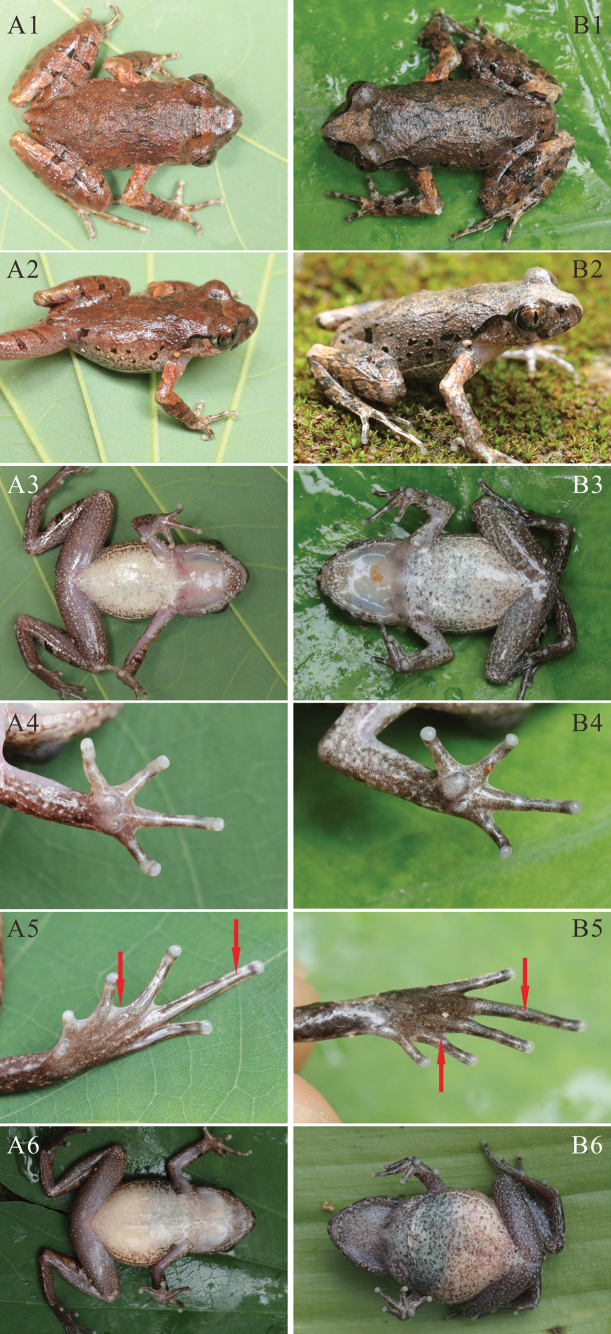
Morphological characters compared between *L.guinanensis* sp. nov. and *L.ventripunctata***A1–6***L.guinanensis* sp. nov. **B1–6***L.ventripunctata***A1, B1** dorsal view **A2, B2** dorsolateral view **A3, B3** ventral view **A4, B4** ventral view of hand **A5, B5** ventral view of foot **A6, B6** ventral view of the gravid female.

Table 2 presents a concise overview of the diagnostic morphological characters of species found north of the Isthmus of Kra. *Leptobrachellaguinanensis* sp. nov. can clearly be distinguished from its phylogenetically close congeners, *L.ventripunctata*. *Leptobrachellaguinanensis* sp. nov. differs from *L.ventripunctata* by a larger body size (SVL 30.5–32.5 mm in males; 38.7–41.8 mm in females vs 25.5–28.0 mm in males, 31.5–35.0 mm in females), ventral surface creamy white without brown spots (vs chest and belly creamy white with many scattered brown spots), ventral surfaces of limbs purplish grey (vs ventral surface of limbs grey-brown with dark brown and white speckling or dots), 1/3 toe webbing and toe lateral fringes wide (vs no toe webbing and lateral fringes), dermal ridges distinct under toes (vs absent) (Fig. 7), tibia-tarsal articulation reaching the centre of eye (vs the level between tympanum and posterior of eye), heels not meeting when thighs are appressed at right angles to body (vs heels overlapping). In addition, *L.guinanensis* sp. nov. differs from *L.ventripunctata* by relatively high dominant frequencies (7.3–8.3 kHz vs 6.1–6.4 kHz), call durations (mean 25.5 ms, ranging 23–31 ms vs mean 145 ms, ranging 65–430 ms) and call intervals (mean 91.2 ms, ranging 55–133 ms vs mean 134 ms, ranging 31–416 ms) (Table 4). Secondly, *L.guinanensis* sp. nov. can be easily distinguished from its sympatric species, *L.shangsiensis*, *L.shiwandashanensis*, and *L.sungi*. *Leptobrachellaguinanensis* sp. nov. differs from *L.shangsiensis* by a larger body size (SVL 30.5–32.5 mm in males, 38.7–41.8 mm in females vs 24.9–29.4 mm in males, 30.8–35.9 mm in females), crossbars of hindlimbs with tubercles (vs lack of tubercles on crossbars of hindlimbs), 1/3 toe webbing (vs toe webbing rudimentary), head width less than length (HW/HL = 0.93 vs HW/HL = 1.15), eye diameter near equal to snout length (ED/SNT = 0.99 vs ED/SNT = 0.78), a pair of glands under the vent (vs absent glands under the vent), dominant frequencies (7.3–8.3 kHz vs 5.5–6.5 kHz), call duration (mean 25.5 ms, ranging 23–31 ms vs mean 66.0 ms, ranging 64–69 ms; Table 4). *Leptobrachellaguinanensis* sp. nov. differs from *L.shiwandashanensis* by relatively larger body size (SVL 30.5–32.5 mm in males; 38.7–41.8 mm in females vs 26.8–29.7 mm in males, 33.7–35.9 mm in females), 1/3 toe webbing and wide lateral fringes on toe (vs no webbing and no lateral fringes on toe), tibia-tarsal articulation reaching to the centre of eye (vs posterior of eye), a pair of glands under the vent (vs absent glands under the vent), dominant frequencies (7.3–8.3 kHz vs 5.3–5.7 kHz), call duration (mean 25.5 ms vs mean 226.6 ms; Table 4). *Leptobrachellaguinanensis* sp. nov. differs from *L.sungi* by conspicuously smaller body size (SVL 30.5–32.5 mm in males; 38.7–41.8 mm in females vs SVL 48.3–52.7 mm in males, 56.7–58.9 mm in females); iris bicoloured, upper half light copper, transitioning to silver in lower half (vs uniform gold green iris), finger II longer than finger I (vs finger I and II equal in length), tympanum distinct and rounded (vs indistinct), dorsal surface brown, an inverted triangle marking between eyes, irregular markings on shoulder and the rear of back (vs dorsum uniformly light brown or with light spots), dominant frequencies (7.3–8.3 kHz vs 2.0–2.7 kHz), call duration (mean 25.5 ms vs mean 59.4 ms), call intervals (mean 91.2 ms vs mean 478.4 ms) (Table 4).

Finally, *L.guinanensis* sp. nov. can be differentiated from other species in the genus *Leptobrachella* based on distinctive bioacoustics and morphological diagnostic characters (for details see Table 2, Suppl. material 1: table S4), as well as genetic divergences (Suppl. material 1: table S3).

## ﻿Discussion

In recent years, five new *Leptobrachella* species have been discovered in the region of Guangxi: *L.damingshanensis* Chen, Yu, Cheng, Meng, Wei, Zhou & Lu, 2021, *L.maoershanensis* (Yuan, Sun, Chen, Rowley & Che, 2017), *L.shangsiensis*, *L.shiwandashanensis*, and *L.wuhuangmontis* Wang, Yang & Wang, 2018 (AmphibiaChina 2023). In addition to these five species, previous studies had identified four additional *Leptobrachella* species: *L.alpina* (Fei, Ye & Li, 1990), *L.bourreti* (Dubois, 1983), *L.liui* (Fei & Ye, 1990), and *L.sungi* (Fei et al. 2012; Mo et al. 2014). Including the newly herein described *L.guinanensis* sp. nov. this elevates the known number of *Leptobrachella* species in the region of Guangxi to at least 10. Additionally, *L.ventripunctata*, originally found in Zhushihe, Mengla County, Xishuangbanna Dai Autonomous Prefecture, Yunnan, China, is widely distributed in southern Yunnan, Guizhou, China, Laos, northern Vietnam, and northern Thailand (Fei et al. 1990, 1992, 2012; Li et al. 2016; Chen et al. 2018; Luong et al. 2019; Wu et al. 2021; Frost 2023) and also Guangxi. However, this is the first recorded sighting of *L.ventripunctata* in Guangxi. The JZS specimens were identified as *L.ventripunctata* based on molecular data and morphological characters. The original diagnostic characters of *L.ventripunctata* include a relatively small body size (SVL 25.5–28.0 mm in males), absence of webbing and lateral fringes on the toes, creamy white chest and belly with scattered brown spots, relatively short hindlimbs, and tibiotarsal articulation reaching between the tympanum and the corner of the eye (Fei et al. 1990, 1992). These diagnostic characters were found to match those of the JZS specimens. However, *L.guinanensis* sp. nov. has 1/3 webbing on the toes, wide lateral fringes on the toes, no spots on the chest and belly, and tibiotarsal articulation reaching the centre of the eye, which are inconsistent with the diagnostic characters of *L.ventripunctata*. The genetic divergences between the new species and *L.ventripunctata* from the type locality are 1.6–2.4%, which is similar to the genetic divergences observed in other comparisons such as *L.brevicrus* Dring, 1983 vs *L.itiokai* Eto, Matsui & Nishikawa, 2016 (1.2%), *L.bijie* Wang, Li, Li, Chen & Wang, 2019 vs *L.oshanensis* (Liu, 1950) (1.7%), *L.jinshaensis* Cheng, Shi, Li, Liu, Li & Wang, 2021 vs *L.purpuraventra* Wang, Li, Li, Chen & Wang, 2019 (1.8%), *L.bijie* vs *L.chishuiensis* Li, Liu, Wei & Wang, 2020 (2.0%), and *L.bijie* vs *L.jinshaensis* (2.0%) (Suppl. material 1: table S3). Additionally, *L.guinanensis* sp. nov. exhibits high dominant frequencies of 7.3–8.3 kHz, which are the highest dominant frequencies ever known in the genus *Leptobrachella* (Suppl. material 1: table S4). Currently, there are no available advertisement calls of *L.ventripunctata* from the type locality. However, the advertisement calls from Yingjiang County, Yunnan, China, which is near the type locality, resemble those of the JZS specimens in terms of call durations, call intervals, and dominant frequency. It is important to note that the advertisement calls of the new species do not overlap with those of the JZS specimens, indicating reproductive isolation between them (Köhler et al. 2017).

*Leptobrachellasungi* is primarily found in northern Vietnam, specifically in the provinces of Vinh Phuc, Yen Bai, Lao Cai, Dien Bien, Phu Tho, Son La, and Tuyen Quang, as well as in Guangxi, China (Frost 2023). Previous studies have indicated that *L.sungi* was only observed in the SWDS area of Guangxi (Fei et al. 2012; Mo et al. 2014). In our current study, we have identified a new range of *L.sungi* in Guangxi, specifically in the Sishuihe Nature Reserve, located in Lingyun County, Guangxi, China (SSH, Fig. 1).

The reserve harbors four different species of *Leptobrachella*, indicating a remarkably high species diversity within the genus. The four species were found in the evergreen forest at SWDS between 400–600 m. However, *L.guinanensis* sp. nov. was found near a stream that was ~ 0.5–1.0 m wide and had running currents. *Leptobrachellashangsiensis* and *L.shiwandashanensis* occur syntopically, but the former tended to call on rocks or near (~ 1.0 m) rocky streams with fast currents, while the latter called near rocky streams ~ 2.0–3.0 m away. *Leptobrachellasungi* was found to call near rocky streams that were ~ 2.0–3.0 m wide with slower currents. The breeding seasons for these species are as follows: *L.guinanensis* sp. nov. breeds in June, *L.shangsiensis* and *L.shiwandashanensis* in April, and *L.sungi* in July. Further research is required to understand how these four sympatric species interact and adapt to their respective niches within the reserve.

## Supplementary Material

XML Treatment for Leptobrachella guinanensis

## ﻿Additional information

### Author ORCIDs

Wei-Cai Chen https://orcid.org/0000-0002-2398-4079

Peng Li https://orcid.org/0000-0001-8311-0544

Wan-Xiao Peng https://orcid.org/0000-0001-5635-9061

You-Jun Liu https://orcid.org/0000-0001-7285-4943

Yong Huang https://orcid.org/0000-0002-3493-9468

### Data availability

All of the data that support the findings of this study are available in the main text or Supplementary Information.

## Supplementary materials

Supplementary material 1 Morphological data Data type xlsx Explanation note **table S1**: References for morphological characters for congeners of the genus *Leptobrachella*. **table S2**: Comparative material examined. **table S3**: Uncorrected p-distance in *Leptobrachella* species based on 16S gene fragments. **table S4**: Dominant frequency of advertisement calls of species available in the genus *Leptobrachella*. **table S5**: Measurements of adult specimen of *L.guinanensis* sp. nov. and *L.ventripunctata*. https://binary.pensoft.net/file/990968This dataset is made available under the Open Database License (http://opendatacommons.org/licenses/odbl/1.0/). The Open Database License (ODbL) is a license agreement intended to allow users to freely share, modify, and use this Dataset while maintaining this same freedom for others, provided that the original source and author(s) are credited.Wei-Cai Chen, Peng Li, Wan-Xiao Peng, You-Jun Liu, Yong Huang

Supplementary material 2 Advertisement calls of *L.sungi* Data type tif https://binary.pensoft.net/file/990969This dataset is made available under the Open Database License (http://opendatacommons.org/licenses/odbl/1.0/). The Open Database License (ODbL) is a license agreement intended to allow users to freely share, modify, and use this Dataset while maintaining this same freedom for others, provided that the original source and author(s) are credited.Wei-Cai Chen, Peng Li, Wan-Xiao Peng, You-Jun Liu, Yong Huang
