# Supplementary figures and images for: ﻿The fourth species of Leptobrachella (Anura, Megophryidae) found at Shiwandashan National Nature Reserve, Guangxi, China
Source: Zookeys. 2024 Feb 22;1192:257–79. doi: 10.3897/zookeys.1192.98352 (PMC10905621; doi:10.3897/zookeys.1192.98352)

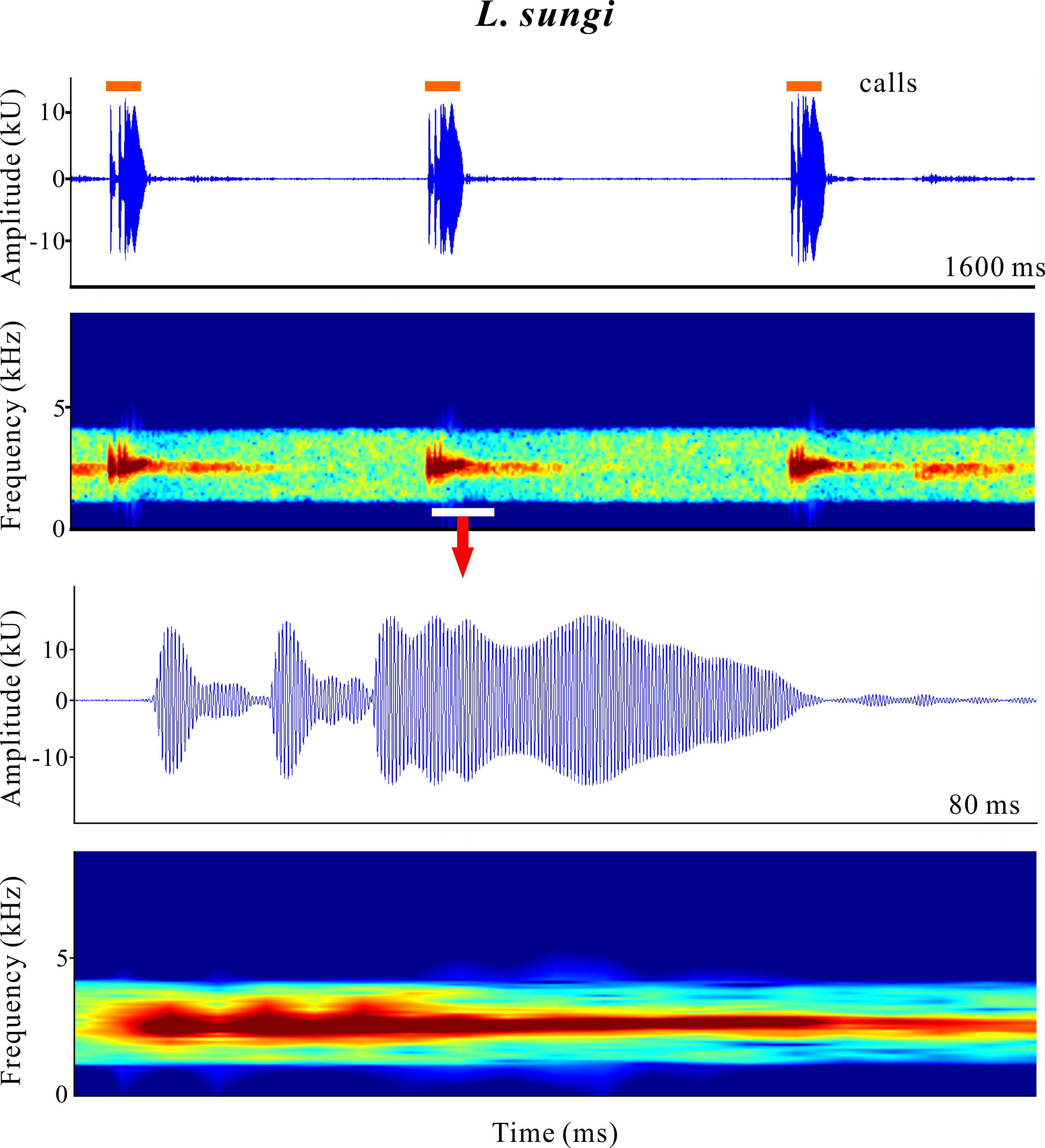

Supplement: Supplementary material 2 — Advertisement calls of L.sungi [file zookeys-1192-257_article-98352__-s002.tif]
